# Increased Protein Encapsulation in Polymersomes with Hydrophobic Membrane Anchoring Peptides in a Scalable Process

**DOI:** 10.3390/ijms22137134

**Published:** 2021-07-01

**Authors:** Michael Mertz, Kathrin Castiglione

**Affiliations:** Institute of Bioprocess Engineering, Friedrich-Alexander-Universität Erlangen-Nürnberg, 91052 Erlangen, Germany; michael.mertz@fau.de

**Keywords:** hydrophobic peptide, polymersome, protein encapsulation, polymer vesicle, functionalization

## Abstract

Hollow vesicles made from a single or double layer of block-copolymer molecules, called polymersomes, represent an important technological platform for new developments in nano-medicine and nano-biotechnology. A central aspect in creating functional polymersomes is their combination with proteins, especially through encapsulation in the inner cavity of the vesicles. When producing polymersomes by techniques such as film rehydration, significant proportions of the proteins used are trapped in the vesicle lumen, resulting in high encapsulation efficiencies. However, because of the difficulty of scaling up, such methods are limited to laboratory experiments and are not suitable for industrial scale production. Recently, we developed a scalable polymersome production process in stirred-tank reactors, but the statistical encapsulation of proteins resulted in fairly low encapsulation efficiencies of around 0.5%. To increase encapsulation in this process, proteins were genetically fused with hydrophobic membrane anchoring peptides. This resulted in encapsulation efficiencies of up to 25.68%. Since proteins are deposited on the outside and inside of the polymer membrane in this process, two methods for the targeted removal of protein domains by proteolysis with tobacco etch virus protease and intein splicing were evaluated. This study demonstrates the proof-of-principle for production of protein-functionalized polymersomes in a scalable process.

## 1. Introduction

Via the self-assembly in aqueous solution, block-copolymers can form hollow, spherical vesicles, called polymersomes [1]. These vesicles are composed of a polymer membrane of a single or double layer of short linear polymer molecules. Triblock-copolymers of the ABA type, in which the outer blocks (A) are hydrophilic and the inner (B) block is hydrophobic, form vesicles with a single layer, in which the linear block-copolymer chains arrange themselves alongside in order to minimize exposure of the central hydrophobic block to the aqueous surrounding [2]. Next to the polymers arranged in parallel (I-shape) some can exist in a U-shape conformation, in which both hydrophilic (A-blocks) are located on the same side of the membrane [3]. In curved membranes, this conformation could occupy more space on one side of the membrane following the membrane curvature. One type of ABA triblock-copolymer is PMOXA-PDMS-PMOXA (poly(2-methoxazoline)-*b*-poly(dimethylsiloxane)-*b*-poly(2-methoxazoline)). It has been used in a number of polymersome designs, which combined the polymersomes with proteins, creating functionalized polymersomes (e.g., [4,5,6,7,8]). The similar fluidity of the central PDMS block to natural lipid membranes enables the functional integration of membrane proteins in PMOXA-PDMS-PMOXA polymersomes [9,10]. The outer PMOXA blocks display low protein binding properties and are biocompatible [11], making this block-copolymer an interesting material for biotechnological and, in particular, medical applications.

Since the early studies on polymersomes, their application in the field of (nano)-medicine has been subject to investigation. Early studies started with the encapsulation of drug molecules, like paclitaxel, doxorubicin, or pravastatin in polymersomes for the targeted treatment of cancer tissue or arteriosclerosis, respectively [12,13]. Another concept to use polymersomes in therapy has been not to encapsulate drug molecules as payload, but instead to include an enzyme for prodrug activation. For example, an encapsulated nucleoside hydrolase from *Trypanosoma vivax* was able to activate the prodrug 2-fluoroadenosine, resulting in the release of cytotoxic 2-fluoroadenine. This polymersome included, next to the nucleoside hydrolase, membrane channel proteins within the polymer membrane to increase mass transport of prodrugs and drugs in and out of the polymersome [5]. A similar concept used polymersomes encapsulating penicillin acylase to synthesize antibiotics on site [6]. A recent example of the potential of polymersomes in nano-medicine was displayed in a vaccine formulation against the Severe Acute Respiratory Syndrome Coronavirus 2 (SARS-CoV2). In this study polymersomes presented the SARS-CoV2 spike protein and showed promising results in first studies in mice [14].

These are only some exemplary studies, showing the potential use of polymersomes for medical purposes. However, to date, only vesicles based on lipid membranes (liposomes) have been approved and are used in pharmaceutical and commercial products [15]. A central aspect to enable the wide range use of polymersomes in commercial products is large-scale production. Recently our group established a polymersome formation process in stirred-tank reactors (STR), which can be scaled based on the defined flow characteristics in STRs [16]. Large-scale production of polymersomes is one part but another aspect is formation of functionalized polymersomes. Many polymersome formulations combine polymersomes with proteins, in particular by encapsulation of enzymes within the polymersome lumen. However, the encapsulation efficiency largely depends on the method of polymersome formation, the chemical properties of the used copolymers and even on the properties of the proteins themselves, leading to largely varying degrees of encapsulation, with reported values ranging between <1% and 100% [8,17,18,19,20]. The formation of polymersomes in STRs is done by injecting the polymer in organic solvent into the aqueous phase. The solvent-switch under controlled stirring leads to the generation of uniform vesicles [21]. Protein to be encapsulated is dissolved in the aqueous phase and ends up within the polymersomes by entrapment of parts of the aqueous phase, which then becomes the lumen (inner compartment) of the polymersome. This means, however, that the encapsulation efficiency is limited by the encapsulated volume. In the case of the PMOXA_15_-PDMS_68_-PMOXA_15_ block copolymer used for polymersome production in the scalable process and in this study, the inner volume is only 0.53% [16]. If other effects arising from the protein come into play in addition, the encapsulation efficiency can be even lower. For example, when encapsulating the enzyme *N*-acetylglucosamine 2-epimerase in this process only 0.36% of the enzyme were entrapped within the polymersomes, which corresponds to only four molecules per vesicle [8].

The bottleneck of statistical encapsulation can be overcome by creating a targeted interaction between polymersomes and proteins. The great efficiency of this concept has already been shown by the immobilization of proteins on the surface of preformed polymersomes, which were concentrated on the polymer membrane by means of hydrophobic membrane anchors [22]. Notably, by genetic fusion of C-terminal hydrophobic peptides to enhanced green fluorescent protein (eGFP) a total coverage of the outer polymersome surface was achieved. In case of polymersomes made of PMOXA_15_-PDMS_68_-PMOXA_15_ this came up to 2320 eGFP molecules per polymersome with a number-based mean diameter of 110 nm [22]. If this concept could also be used to direct proteins to the inner side of the membrane, the encapsulation efficiency could be significantly increased. Although the surface of the inner membrane is smaller it would theoretically still allow for up to 1027 eGFP per polymersome (see Supplement S1). For these reasons, in this study we evaluated different hydrophobic membrane anchoring peptides regarding their potential to increase protein encapsulation in PMOXA_15_-PDMS_68_-PMOXA_15_ polymersomes. Additionally, we looked at molecular tools to specifically release protein domains from immobilized proteins on the surface of polymersomes, via proteolysis by tobacco etch virus (TEV) protease and inclusion of an intein domain.

## 2. Results

### 2.1. Protein Encapsulation in Polymersomes with Hydrophobic Membrane Anchoring Peptides

**Figure 1:**
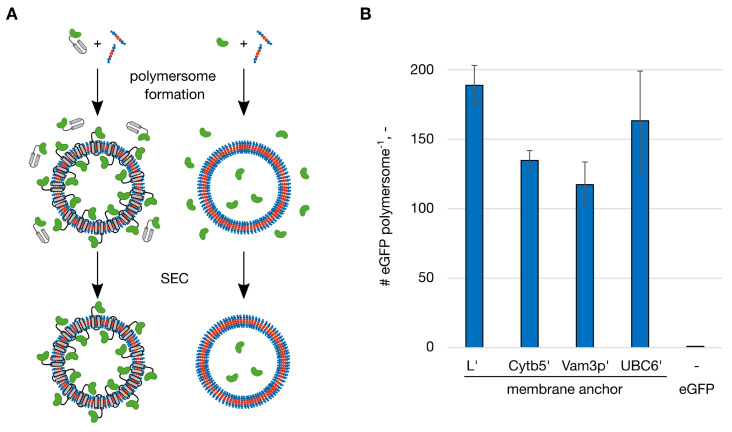
Functionalization of polymersomes with membrane-anchoring enhanced green fluorescent protein (eGFP). Polymersomes were functionalized with fusion proteins of eGFP and the membrane-anchoring peptides L’, Cytb5′, Vam3p’ and UBC6′ and without a membrane-anchoring peptide (eGFP). (**A**) The proteins with (left) or without (right) membrane anchor were mixed with the amphiphilic polymer (blue: hydrophilic blocks, red: hydrophobic block). The eGFP domains are shown in green, the membrane anchoring domains in grey. After polymersome formation, the dispersions were processed by size-exclusion chromatography (SEC) to separate the functionalized vesicles from unincorporated protein. (**B**) Number (#) of eGFP molecules per polymersome after the functionalization. The proteins were applied at a concentration of 0.50 g/L with the exception of eGFP-UBC6′ (0.40 g/L). Error bars represent the standard deviation in technical triplicates.

To test the functionalization of polymersomes with membrane anchoring proteins, vesicles were formed in presence of fusion proteins of eGFP and C-terminal membrane anchoring domains (hydrophobic peptides). The membrane anchoring peptides originated from rabbit liver cytochrome *b*_5_ (Cytb5′), the lysis protein L from the SM2 phage (L’), the syntaxin Vam3p (Vam3p’) and the ubiquitin conjugating enzyme 6 (UBC6′) from yeast [22]. For comparison eGFP without a membrane anchor was encapsulated. The proteins were applied in concentrations of 0.50 g/L before starting polymersome formation, with the exception of eGFP-UBC6′, which was used at 0.40 g/L due to low protein expression yields. After polymersome formation in miniaturized STRs the dispersions were processed by size-exclusion chromatography (SEC) to separate the functionalized vesicles from unincorporated protein (Figure 1A). Quantification of the eGFP fluorescence intensity as well as the light extinction (optical density) at 350 nm made it possible to quantify the number of eGFPs associated with the polymersomes. In case of eGFP without a membrane anchor only encapsulated proteins were present. When membrane anchoring eGFP were used proteins can be present on the outer and inner membrane surface (Figure 1A). Encapsulation of eGFP using 0.50 g/L protein resulted in an average number of 0.3 eGFP per polymersome. Since polymersome dispersions contain vesicles of multiple sizes (size distribution) the average number of proteins per polymersome as a decimal number is stated. In this case, it means that out of ten polymersomes three are entrapping an eGFP molecule. Formation of polymersomes in presence of fusion proteins with membrane anchors resulted in far higher numbers of proteins per polymersome (Figure 1B). eGFP-L’ presented the highest functionalization with 188.6 ± 14.4 proteins per polymersome.

Based on these numbers, the functionalization efficiencies (FE%) could be calculated as the ratio between the number of proteins associated with polymersomes and the amount of protein used initially. During the polymersome formation in STRs, as done in these experiments, a dispersion of 1% (*w*/*v*) was produced. From the characterization of the polymersomes formed in this process the number of polymersomes per liter of a 1 % (*w*/*v*) dispersion was calculated to 1.83 × 10^16^ L^−1^ [21]. From the application of 0.50 g/L protein (0.40 g/L for eGFP-UBC6′) the FE% result in 0.05% for eGFP, 43.1% for eGFP-L’, 29.0% for eGFP-Cytb5′, 23.2% for eGFP-Vam3p’ and 39.7% for eGFP-UBC6′. This showed very clearly how the introduction of the hydrophobic membrane anchor increased the incorporation of protein into polymersomes. Whereas the entrapment of eGFP without membrane anchor was limited, primarily by the low entrapped volume, a domain enabling affinity to the forming polymer membrane/polymersome overcame this limitation. As mentioned in the introduction the inner volume for polymersomes formed in STRs with the PMOXA_15_-PDMS_68_-PMOXA_15_ block-copolymer is 0.53%, which therefore represents the maximal FE% by statistical entrapment of proteins. The lower FE% found for eGFP could arise from unfavorable interactions between the protein and the copolymer during polymersome formation in the STRs. eGFP variants with membrane anchors on the other hand showed FE% between 44 and 81 times higher than the maximum of 0.53 %.

**Figure 2:**
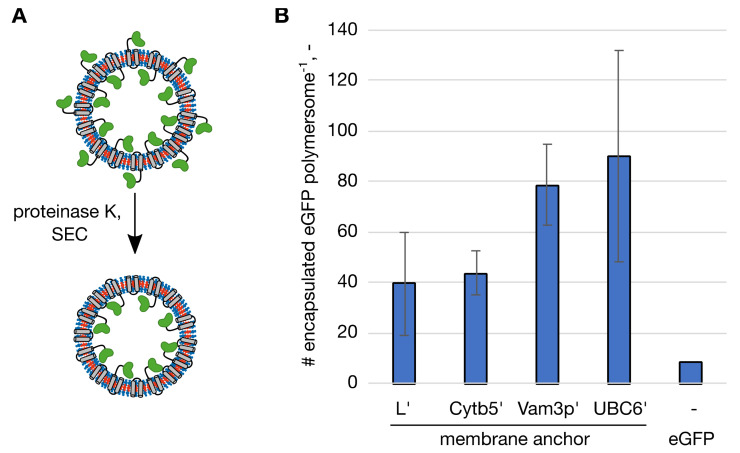
Maximal numbers of encapsulated enhanced green fluorescent protein (eGFP) molecules per polymersome with the respective membrane anchor and soluble eGFP without a membrane anchor as a control. (**A**) Polymersomes with membrane-anchoring eGFP were subjected to proteinase K digest and processed by size exclusion chromatography (SEC) to separate the polymersomes from the protein set free from the outer surface. The eGFP domains are shown in green, the membrane anchoring domains in grey. (**B**) Number (#) of encapsulated eGFP molecules per polymersome. Highest encapsulation was reached at different initial protein concentrations, L’: 0.75 g/L, Cytb5′: 0.78 g/L, Vam3p’: 0.30 g/L, UBC6′: 0.40 g/L and eGFP: 4.30 g/L. The standard deviation in technical triplicates is indicated by the error bars.

To see if using the membrane anchors to enhance not only functionalization but foremost protein encapsulation, we had to analyze the number of proteins present within the polymersome lumen. This meant to distinguish between the proteins anchored to the outer polymersome surface and the ones immobilized on the inner membrane surface. To have a clear distinction when describing the membrane anchored protein located on the inner versus the outer membrane surface of the polymersomes, proteins contained within a polymersome are referred to as encapsulated protein throughout this manuscript. Since PMOXA-PDMS-PMOXA polymersomes are impermeable for large biomolecules such as proteins [7], we used proteinase K to digest the proteins present on the outer surface (Figure 2A). This fairly unspecific protease had been used to digest proteins on the outer surface of polymersomes before [23,24]. A control experiment in which proteins were immobilized to the outer surface of the polymersomes demonstrated that proteinase K efficiently removed proteins present on the outer polymersome surface (Figure S2). Following the proteinase K digest polymersome samples were again processed by SEC, to separate the vesicles from the protein set free from the outer surface and encapsulated eGFP was quantified from the fluorescence intensity. To find maximal protein encapsulation, polymersomes were formed in presence of a range of protein concentrations of the membrane anchoring eGFP, resulting in maximal numbers of 39.4 ± 20.5 eGFP per polymersome for eGFP-L’, 43.6 ± 8.4 for eGFP-Cytb5′, 78.3 ± 16.1 for eGFP-Vam3p’ and 89.8 ± 41.8 for eGFP-UBC6′ (Figure 2B). These maximal numbers for the respective membrane anchoring peptides were reached when applying 0.75 g/L eGFP-L’, 0.78 g/L eGFP-Cytb5′, 0.30 g/L eGFP-Vam3p’ or 0.40 g/L eGFP-UBC6′. Considering the inner volume of the polymersomes, these numbers are equivalent to protein concentrations between 8.31 g/L (eGFP-L’) and 14.85 g/L (eGFP-UBC6′) within the polymersomes. The number of proteins encapsulated at other protein concentrations as well as the total number of proteins per polymersome functionalized are presented in the Supplement S3. As a reference, polymersomes were formed in presence of eGFP without membrane anchor at increasing concentrations as well. The highest tested concentration was 4.30 g/L, which encapsulated an average number of 8.7 eGFP per polymersome (Figure 2B).

Knowing the number of encapsulated proteins per polymersome made it possible to calculate the encapsulation efficiencies (EE%) for the membrane anchoring peptides. EE% was calculated as the ratio of encapsulated protein to total amount of protein before polymersome formation. In case of eGFP, where all proteins are encapsulated, since eGFP does not adsorb on the outer surface of the polymersomes [22], EE% and FE% are essentially the same, resulting in 0.18% at 4.30 g/L. Although this value was higher when 4.30 g/L protein were used than for 0.50 g/L (0.05%), it is still only about 1/3 of the maximal encapsulation efficiency for statistical encapsulation (0.53%, equivalent to the percentage of encapsulated volume). With membrane anchors protein encapsulation was more efficient with values of 5.87% (eGFP-L’), 6.00% (eGFP-Cytb5′), 25.68 % (eGFP-Vam3p’) and 19.67 % (eGFP-UBC6′) at the respective protein concentrations that gave the highest numbers of encapsulated proteins. As seen before for the protein concentrations at which the highest number of proteins were encapsulated, EE% showed differences between the two groups of membrane anchors. The longer and moderately hydrophobic L’ and Cytb5′ anchors reach maximal encapsulation at higher protein concentrations than the shorter and more hydrophobic Vam3p’ and UBC6′, which in turn resulted in higher EE% for Vam3p’ and UBC6′.

When comparing the distribution of proteins on the surface of polymersomes with respect to their inwards or outwards facing orientation, differences between the longer and moderately hydrophobic peptides (L’ and Cytb5′) and the shorter hydrophobic peptides (Vam3p’ and UBC6′) can be seen. Whereas L’ and Cytb5′ membrane anchors preferred the outward facing orientation, the opposite was the case for the shorter and highly hydrophobic peptides Vam3p’ and UBC6′. So far, no clear explanation can be given for this effect on polymer membrane, however, some results from computational studies on membrane-penetrating peptides in lipid membranes point towards influences of penetration depth of the peptides and effects of protein clustering on membrane curvature [25,26]. Further, effects of the hydrophobicity of the peptides on the promotion of positive membrane curvature by less hydrophobic and negative membrane curvature by more hydrophobic peptides were noted [27]. It must be kept in mind, however, that these studies were done on lipid membranes.

Dynamic light scattering (DLS) is a robust method to characterize polymersome dispersions. It was used to see if and how polymersomes are affected, when formed in the presence of protein and especially of those with membrane anchoring domains. Since the latter proteins have a domain interacting with the polymer membrane, effects on the formed polymersomes were possible. The intensity weighted harmonic mean diameter, z-average, of polymersomes formed in buffer without proteins was 209.8 ± 1.7 nm. When soluble eGFP was present the z-average increased to 217.2 ± 0.9 nm, indicating an influence of the presence of proteins on polymersomes (Table 1). Next to the z-average, the size-distribution of the created vesicle dispersion is of interest. This is represented by the polydispersity index (PDI), which is defined as a number between 0 and 1 with 0 representing a sample of uniform particles and 1 a heterogeneous particle size distribution. Without proteins polymersomes showed a PDI of 0.224 ± 0.017, which increased slightly in presence of eGFP to 0.241 ± 0.004, analogous to the change in z-average. A bigger change was present, when polymersomes were formed in presence of eGFP with membrane anchors. Here, PDI values were between 0.375 ± 0.037 and 0.395 ± 0.049 (Table 1). The z-average of polymersomes formed in the presence of eGFP with membrane anchors was also increased compared to polymersomes formed without protein and with eGFP (Table 1). When considering the presence of immobilized eGFP on the outside of these polymersomes, an increase in vesicle size can be expected. However, a complete layer of eGFP should only cause an increase in diameter of around 10 nm (see Supplement S1). Here, the z-average was increased between 27.4 and 41.4 nm, which is well above this number. Although a slight tendency of increasing vesicle size with the number of proteins located on the outside of polymersomes can be noted (Table 1), the difference between polymersomes with the highest number (eGFP-L’) and the lowest (eGFP-Vam3p’) are not significant in an unpaired *t*-test (p = 0.118). Further, considering the increased polydispersity of the polymersomes with membrane anchoring proteins (Table 1) this larger than expected increase could be influenced by the higher light scattering intensity of larger particles in comparison to smaller ones.

Additionally, we compared the different membrane-anchored constructs from a practical perspective. If a protein should be encapsulated, not only the number of proteins in the vesicle lumen should be considered, but also their correct folding and the relative effort to produce the individual constructs in larger quantities. In the case of eGFP, the specific fluorescence compared to the protein without anchor domain can provide an indication of correct folding and unhindered chromophore formation. If all these factors are equally weighted, the best anchor is deduced from the mathematical product of relative fluorescence, expression yield and encapsulation efficiency at the optimum loading in the vesicle interior. These results are summarized in Table 2.

Table 2 shows that, according to this calculation, the construct with the L’ anchor is the most suitable, although the actual EE% is the lowest, since the anchor has no negative influence on protein folding and showed the highest expression yield. When strictly comparing the numbers the Vam3p’ anchor is closely behind, by compensating for the negative influence on chromophore formation through the excellent EE%. The UBC6′ anchor had the biggest negative impact on protein folding, which might also explain the low expression yield. These two drawbacks could not be compensated by the high EE%, resulting in the lowest suitability. Whether a slightly distorted fold can be tolerated for a protein to be encapsulated is a decision that has to be made on a case-by-case basis. If, for example, enzymatically active nanoreactors are to be created, very good results could also be achieved with anchors that influence the folding. In the case of immobilized enzymes, it often happens that the catalytic activity decreases due to the connection to a support. On the other hand, there are scenarios in which a protein that is incorrectly folded cannot be tolerated—for example if it is supposed to interact with other proteins or if the activity per protein must be at its maximum. In addition, it must be emphasized that the effect of the anchor domains on different fusion partners cannot be generalized, which is why, ideally, several anchors should be compared for a protein to be encapsulated to select the best fusion protein.

### 2.2. Specific Release of Immobilized Protein Domains from the Surface of Polymersomes

When using proteins with membrane anchoring domains during polymersome formation, the process of membrane insertion does not differentiate between the outer and inner membrane of the vesicle. Due to symmetry of the ABA triblock-copolymer both inner and outer surface have the same chemical properties (PMOXA blocks) and experience only some physical differences with respect to the surface curvature.

As we have seen in the encapsulation experiments, proteins are immobilized on both, the inner and outer surfaces. For protein encapsulation this means that a certain ratio of the proteins is oriented in the ‘wrong’ or undesired orientation. In this case, it would be on the outer surface. If general polymersome functionalization is the primary task, this does not pose an issue. For more specialized designs of functional vesicles, however, positioning of proteins can be crucial. For example, protein A could interfere in its function with protein B, which must be positioned on the outside of the polymersome. Additionally, if proteins are already present on the polymersome surface, the immobilization of further proteins could be impeded.

For these reasons, we incorporated different molecular tools to enable a specific release of immobilized protein domains from the polymersome surface. The strategy to remove proteins on the outer surface with an unspecific protease like in the proteinase K digest used for analysis of protein encapsulation, could have damaging effects on proteins like membrane channels, which might also be incorporated into the polymersomes during polymersome formation. For targeted removal of protein domains, the protease from TEV as well as the gyrase A intein domain from *Mycobacterium xenopi* were considered. The TEV protease has a high sequence specificity towards the ENLYFQ|G/S motif, cutting between glutamine and glycine/serine [28,29]. It can be used at a broad range of temperatures and pH [30,31]. The intein domain has a natural function to cut itself out of the precursor protein, thereby joining the protein domain upstream and downstream of the intein together [32]. It can be genetically modified so that the upstream (N-terminal) domain can be separated from the intein and the remaining protein by addition of nucleophilic substances like thiols [33,34].

When choosing a suitable construct for the further experiments, we looked at the Cytb5′ membrane anchor. The reason for this is that this membrane anchor has proven itself as an efficient candidate for the immobilization of proteins on the surface of polymersomes [22], which was a prerequisite for the targeted removal of proteins at the outer surface of the polymersomes during the experiment.

**Figure 3:**
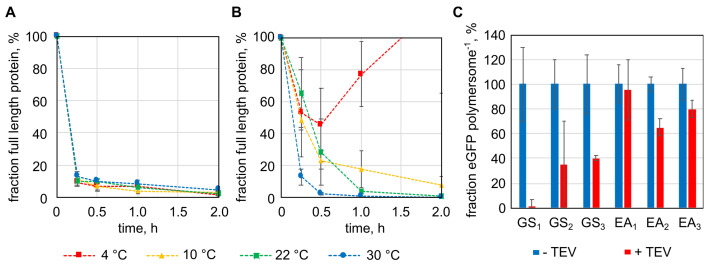
Targeted proteolytic release of protein domains from the surface of polymersomes by tobacco etch virus (TEV) protease. (**A**) and (**B**): proteolysis of enhanced green fluorescent protein (eGFP) with TEV protease recognition sequences and the Cytb5′ membrane anchoring peptide (A: eGFP-GS_2_/TEV-Cytb5′, B: eGFP-EA_2_/TEV-Cytb5′) in solution. (**C**): TEV protease proteolysis of immobilized fusion proteins of eGFP, the TEV recognition sequence flanked by different linkers (EA: EAAAK-linker, GS: GSSSS-linker, numbers indicate repeats of the EA or GS peptide) and the Cytb5′ membrane anchor. Error bars represent the standard deviation in technical triplicates.

The TEV protease recognition sequence (ENLYFQS) was cloned into the gene of the eGFP fusion protein with the Cytb5′ membrane anchor, between the *egfp* gene and the sequence of the deca-alanine linker (*pA*) (*egfp-pA-cytb5′*). Flanking the TEV protease recognition sequence two repetitions of a flexible (GSSSS; GS_2_) or rigid (EAAAK; EA_2_) linker were included, resulting in the fusion proteins eGFP-GS_2_/TEV-Cytb5′ and eGFP-EA_2_/TEV-Cytb5′. Proteolysis of the two proteins by the TEV protease was tested at temperatures between 4 and 30 °C (Figure 3A,B). The eGFP-GS_2_/TEV-Cytb5′ construct was processed efficiently by the TEV protease, reducing the full-length protein to around 10% in 15 min (first probed time point) at all tested temperatures (Figure 3A). The fusion protein with the rigid linker eGFP-EA_2_/TEV-Cytb5′ was influenced to a greater extent by the present temperature (Figure 3B). At 30 °C, the amount of full-length protein was reduced to 10%, similar to eGFP-GS_2_/TEV-Cytb5′ but at 22 and 10 °C one and two hours, respectively, were necessary to digest more than 90% of the full-length protein. At 4 °C no coherent results were obtained (Figure 3B), indicating that proteolysis of eGFP-EA_2_/TEV-Cytb5′ should be done at temperatures between 10 and 30 °C. After showing proteolysis in solution, fusion proteins with linkers of different lengths, one to three repeats of the GS or EA sequences, were cloned and immobilized to the outer surface of polymersomes. Proteolysis with TEV protease was done overnight at 22 °C (room temperature) (Figure 3C). As seen in the previous experiments, constructs with the flexible linker were cut more efficient, removing between 59.6 and 98.9% of the immobilized protein from the polymersomes. When the rigid linker was part of the fusion protein only 4.2 to 35.0% of the proteins were removed (Figure 3C).

Next to the TEV protease the feasibility of using an intein for specific removal of immobilized protein domains was elucidated. In contrast to the TEV protease, which as an enzyme is too large to cross the polymer membrane, uncharged small molecules can diffuse across the polymer membrane [35]. Since intein splicing is induced by the addition of thiols like dithiothreitol (DTT), 2-mercaptoethanol (2ME) or cysteine (cys), a release of the immobilized protein domain should be possible within the polymersome lumen by including the intein domain. DTT has been shown to diffuse across cellular membranes [36,37]. Experiments with PMOXA-PDMS-PMOXA polymersomes did not allow the diffusion rate to be determined but a qualitative assessment that confirmed the diffusion of DTT across the polymer membrane within minutes or even seconds (Supplement S4).

**Figure 4:**
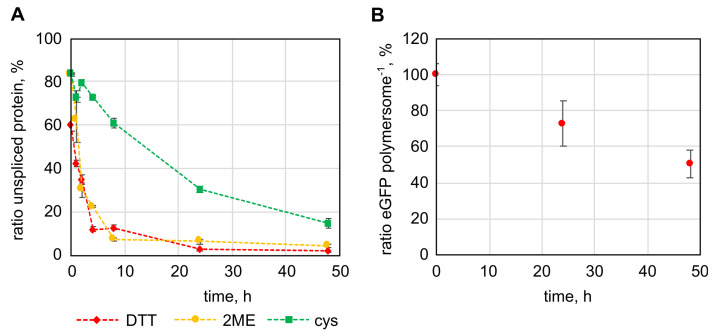
Intein-splicing mediated release of immobilized protein domains from the surface of polymersomes. (**A**): Splicing of the fusion protein of enhanced green fluorescent protein (eGFP), the *Mycobacterium xenopi* gyrase A intein and the Cytb5′ membrane anchor in solution by 50 mM dithiothreitol (DTT), 2-mercaptoethanol (2ME) and cysteine (cys). (**B**): Splicing of eGFP-Int(-1N)-Cytb5′ immobilized to the outer surface of polymersomes with 50 mM DTT. The error bars represent the deviation of three parallel experiments using samples originating from the same initial protein solution (**A**) or polymersome dispersion (**B**).

The intein domain (*int*) was cloned between the *egfp* gene and deca-alanine linker, followed by the *cytb5′* membrane anchor sequence, resulting in the *egfp-int-pa-cytb5′* gene. The last amino acid of the intein was mutated to alanine (N198A), enabling cleavage of the full-length protein upon addition of thiols [33,34]. Splicing of the protein was tested with the protein in solution using DTT, 2ME and cys (Figure 4A). DTT and 2ME reduced the amount of full-length protein to around 10% within 8 h, whereas cys spliced the protein at a slower rate, splicing only around 40% of the protein within 8 h and reducing the full-length protein only to about 15% after 48 h. Variation of thiol concentration (between 10 and 100 mM) and temperature (between 4 to 37 °C) showed that DTT at a concentration of 50 mM performed best in induction of intein splicing, followed closely by 2ME (Figure S7). Cys generally induced splicing at a slower rate, even when 100 mM cys were used. The temperature had a rather small influence on intein splicing with DTT and 2ME (Figure S8). Since the amino acid in the position before the intein can have a direct impact on intein splicing, a screening was done. The result was that asparagine caused the most efficient splicing with 50 mM DTT while showing only little unspecific splicing (Figure S9). Having tested splicing of the eGFP-Int-Cytb5′ fusion protein in solution, intein splicing of immobilized proteins was to be examined next. For this purpose, eGFP-Int(-1N)-Cytb5′ was immobilized to the outer surface of polymersomes and DTT was added in 50 mM concentration to induce splicing. After 24 h the number of proteins present on the polymersomes was reduced to 72.7 ± 12.5 % and after 48 h to 50.4 ± 7.7% (Figure 4B). Splicing in the immobilized state proceeded significantly slower but represents still a successful method to release protein domains from the polymersome surface. Although the intein splicing on the outer membrane surface of the polymersomes to release protein domains is the main issue, the ability to release immobilized protein domains within the vesicles would be another feature made possible by the inclusion of the intein domain. However, encapsulation of the fusion protein of all three domains occurred in surprisingly low numbers. As a result, only a maximum of 4.5 proteins per polymersome could be encapsulated at the highest protein concentration that we were able to test (0.45 g/L). Since the structure of the fusion protein could not be predicted by simulation and the intein domain has a special horse-shoe like structure that brings its N- and C-terminus in close proximity to enable its natural function, the structure of the fusion protein could have unpredictable effects on protein encapsulation. This low number of encapsulated proteins did not make it possible to detect the splicing status of encapsulated proteins. Still, since the splicing of the immobilized intein and DTT diffusion into the polymersome were shown, intein splicing within PMOXA-PDMS-PMOXA polymersomes can be regarded as feasible.

A direct comparison of the protein release from the polymersome surface by means of TEV protease and intein domain shows that the TEV protease enables more complete cleavage. The TEV protease could release up to 98.9% of the immobilized protein from the polymersomes in an overnight incubation, while the intein domain was only able to release 49.6% within 48 h. For this reason, the influence of the TEV recognition sequence on protein encapsulation was additionally investigated. As expected, the few extra amino acids showed no negative effect on the encapsulation of eGFP-GS_1_/TEV-Cytb5′in comparison to eGFP-Cytb5′. At a protein concentration of 0.60 g/L 35.1 ± 1.7 eGFP-GS_1_/TEV-Cytb5′ were encapsulated, which is comparable to 28.4 ± 9.4 eGFP-Cytb5′ per polymersome at 0.58 g/L protein.

## 3. Discussion

In this study we looked at the possibility to increase protein encapsulation in polymersomes made from PMOXA_15_-PDMS_68_-PMOXA_15_ block-copolymers produced in a scalable process in STRs. Combining eGFP with hydrophobic membrane anchoring domains enabled the encapsulation of up to 89.8 eGFP per polymersome, which is equivalent to a protein concentration within the polymersomes of 14.85 g/L. Compared to the encapsulation of soluble eGFP by statistical entrapment this represents an increase by 299.3-fold when comparing the numbers obtained at similar protein concentrations (0.40 g/L eGFP-UBC6′ and 0.50 g/L eGFP).

The EE%s obtained in this study range between 5.87% (L’) and 25.68% (Vam3p’) at the protein concentrations resulting in the highest numbers of encapsulated proteins. Other studies have reported EE% values between 0.36% [8] and up to 100% [17] for polymersomes made from PMOXA-PDMS-PMOXA, although with other proteins. In the last study reporting 100% encapsulation of nerve growth factor, film rehydration was used for polymersome preparation. Encapsulation of bovine serum albumin (BSA) in the mentioned study was reported with 48.6% EE% [17], further underscoring that the EE% is influenced by properties of the respective proteins. In comparison to solvent switch methods film rehydration shows generally higher encapsulation efficiencies due to the formation of larger vesicles with a range of shapes that encapsulate higher volumes [38]. Polymersomes made from other block-copolymers than PMOXA-PDMS-PMOXA such as PEG-PLA (poly(lactic acid)) or PEG-PCL (poly(ethylene glycol)-poly(caprolactone)) encapsulating L-asparaginase showed an EE% of 4.9% [39] and up to 20% [19] respectively. The higher EE% of 20 % was also achieved using film rehydration, whereas for encapsulation of L-asparaginase in PEG-PLA polymersomes a temperature induced solubility switch was used. Similar to the principle of the solvent-switch method used in this study, here block-copolymers are designed to change their solubility behavior in accordance to temperature. The temperature change reduces the solubility of the polymer resulting in aggregation and polymersome formation. Another approach using the so-called ‘direct hydration’ produced polymersomes from PEG-PPS (poly(propylene sulfide)) encapsulating bovine γ-globulin, BSA and ovalbumin with EE%s of 15%, 19% and 37%, respectively [20]. Very high numbers of encapsulated proteins were obtained in a recent study by Bueno et al. [40]. Following up on previous work in the same group [41], electroporation was used for protein loading of preformed polymersomes, resulting in impressive numbers of up to 800 enzymes of L-asparaginase per polymersome. Previous preliminary experiments performed in our group showed that electroporation did not work with PMOXA-PDMS-PMOXA polymersomes for protein loading, even though, in addition to the published device settings, a wide range of possible electroporation conditions was also investigated. The polymersomes used by Bueno consisted of a mixture of two block-copolymers, PMPC-PDPA (poly((2-methacryloyl) ethylphosphorylcholine)-poly(2-(diisopropylamino)ethyl methacrylate)) and PEO-PBO (poly(ethyleneoxide)-poly(butadiene)), forming asymmetric polymersomes with the two types of polymers forming membranes in separated locations on the polymersomes. PMPC-PDPA made up the majority of the polymer membrane and PEO-PBP molecules created a small hump on the vesicles. The thickness of the PEO-PBO membrane is with 2 nm thinner than the PMPC-PDPA membrane with 6.8 nm, increasing electroporation efficiency via this membrane patch [40]. The membrane thickness of PMOXA_15_-PDMS_68_-PMOXA_15_ polymersomes is with 14 nm significantly thicker than PMPC-PDPA or PEO-PBO membranes and PMOXA-PDMS-PMOXA bares no charge. Both these effects could be the reason why electroporation was only successful with PMPC-PDPA/PEO-PBO polymersomes. Electroporation is mostly used on a small scale of up to one milliliter although designs for flow electroporation have been brought forward to address scalability of the method [42,43].

Again, usage of different proteins, block-copolymers and polymersome formation methods makes comparison of the resulting EE%s challenging but with 26.68% for the Vam3p’ membrane anchor, reasonable results were obtained with hydrophobic membrane anchoring peptides. All in all, this method, using hydrophobic membrane anchoring peptides, showed how a scalable polymersome formation process, which previously could only yield low EE%s (max. 0.53%) by statistical encapsulation, can be used to form polymersomes with higher protein loading and to increase the EE% to around 1/4 of the applied protein.

Targeted release of immobilized protein domains was studied by including a TEV protease recognition sequence or an intein domain from the gyrase A of *M. xenopi* in the fusion proteins. When it comes to the release of proteins from polymersomes, delivery of the encapsulated proteins has been the subject of investigation in most cases [44,45,46]. Both methods, targeted proteolysis and intein splicing, managed to liberate immobilized protein domains, although proteolysis with TEV protease showed a more complete removal with only 1.1% remaining as opposed to 50.4% in case of the intein. Thus, especially the TEV protease is a useful tool to remove protein domains in the ‘wrong’/outward facing orientation after polymersome formation with membrane anchoring proteins.

## 4. Materials and Methods

### 4.1. Genetic Material and Cloning of Plasmids

Plasmids harboring genes for eGFP and membrane anchoring eGFP (pET28-eGFP, pET28-eGFP-pA-Cytb5′, pET28-eGFP-pA-L’, pET28-eGFP-pA-Vam3p’, pET28-eGFP-pA-UBC6′) were created as part of a preceding study [22]. Genetic sequences coding for the *Mycobacterium xenopi* gyrase A intein as well as GSSSS and EAAAK-linker sequences with TEV protease recognition sequences of two and three linker sequence repeats were synthesized by Eurofins Genomics, Ebersberg, Germany. The sequences of synthesized genes are given in the Supplement S6. Because *Eco*RI sites were present up- and downstream of the *pA* gene, the restriction site upstream of the *pA* sequence in the pET28-eGFP-pA-Cytb5′ plasmid was changed from *Eco*RI to *Bam*HI by changing the *pA* sequence to a GS(GGSG)_2_ sequence, by PCR with the primers eGFP NdeI F and GSGG2 EcoRI R (Table S1) and restriction cloning with *Nde*I and *Eco*RI, resulting in the plasmid pET28-eGFP-GGSG_2_-Cytb5′. Then, the intein gene was cloned into the pET28-eGFP-GGSG_2_-Cytb5′ plasmid, between the *Bam*HI and *Eco*RI restriction sites (sequence of synthesized DNA is given in the Supplement S6, Table S2), thereby restoring the *pA* sequence and introducing a *Not*I restriction site in the first 8 base pairs of the *pA* sequence, creating the plasmid pET28-eGFP-Int-pA-Cytb5′. The linker sequences of one GSSSS or EAAAK repeat and the TEV protease recognition sequence were produced from oligo-nucleotides by annealed oligo cloning (AOC). To 23 µL AOC buffer (10 mM tris-HCl, 50 mM NaCl, 1 mM EDTA, pH 8.0) 1 µL of each primer of the primer pairs AOC GS1 F, AOC GS1 R and AOC EA1 F, AOC EA1 R was added. Primer sequences are listed in Table S1. Hybridization of the oligonucleotides to produce double stranded DNA sequences with single stranded overlaps according to the restriction sites (*Bam*HI and *Not*I) in the target vector (pET28-eGFP-Int-pA-Cytb5′) was done by heating the primers in AOC buffer to 95 °C for 5 min, followed by a cooling phase with a linear profile down to 25 °C over 45 min, in a FastGene Ultra Cycler Gradient thermocycler (Nippon Genetics Europe, Düren, Germany). Linker peptides of two and three repeats were amplified by PCR from synthesized genetic sequences using Q5 DNA polymerase (New England Biolabs, Ipswitch, MA, USA) following the supplier’s instructions. Sequences of the synthesized genes are listed in Table S2 and corresponding primers are listed in Table S1. The PCR products and the pET28-eGFP-Int-pA-Cytb5′ target vector were digested with *Bam*HI and *Not*I (New England Biolabs, Ipswitch, MA, USA) following the supplier’s instructions. The insert DNA fragments originating from DNA synthesis or PCR were ligated into the target vector with T4 DNA ligase (New England Biolabs, Ipswitch, MA, USA) following the supplier’s instructions. DNA fragments from AOC were diluted 1:20 with water and 1 µL thereof was used for ligation with T4 DNA ligase. Sequences of all plasmids were verified by Sanger sequencing (Eurofins Genomics, Ebersberg, Germany).

Saturation mutagenesis of the amino acid preceding the intein was done using the quick-change method. For the PCR, the primers Int-1X F and Int-1X R (Table S1) and the Q5 DNA polymerase (New England Biolabs, Ipswitch, MA, USA) were used following the supplier’s instructions. The parent plasmid was pET28-eGFP-Int-pA-Cytb5′. After confirmation of the appropriate size of the PCR product via agarose gel electrophoresis a *Dpn*I (New England Biolabs, Ipswitch, MA, USA) digest was done, followed by transformation of the genetic material into *E. coli* DH5a (Invitrogen, Carlsbad, CA, USA) bacteria by heat-shock transformation. The mutated plasmids were confirmed by sequencing (Eurofins Genomics, Ebersberg, Germany).

### 4.2. Protein Expression and Purification

Proteins were expressed in *Escherichia coli* BL21 (DE3) cells (Novagen, San Diego, USA), transformed with the appropriate plasmids. A colony was picked from an LB-agar plate, inoculating a preculture in LB medium, supplemented with 50 mg/L kanamycin. The preculture was grown overnight at 37 °C and agitation. From this preculture terrific broth (TB) medium (12 g/L peptone from casein, 24 g/L yeast extract, 4 mL/L glycerol, 12.54 g/L di-potassium hydrogen phosphate, 2.13 g/L potassium di-hydrogen phosphate, pH 7.4) was inoculated with 0.25% (*v*/*v*) of the preculture in cultivation flasks, filled to 20% of their nominal volume. When expressing eGFP without a membrane anchor LB medium was used. The cultures were grown at 37 °C with agitation at 140 rpm, 5 cm orbital motion until an optical density at 600 nm between 0.6 and 0.8 was reached. At this point protein expression was induced by addition of isopropylthiogalactopyranoside to a final concentration of 1 mM. Bacteria were further cultivated at 20 °C with agitation at 120 rpm for 16 to 20 h.

After protein expression, bacteria were pelleted for 15 min at 4,500 xg. For every gram wet cell mass 5 mL immobilized metal affinity chromatography (IMAC) binding buffer (20 mM sodium-phosphate, 500 mM NaCl, 40 mM imidazole, pH 7.5), supplemented with 0.1 g/L lysozyme and 1 mM phenylmethylsulfonyl fluoride were added and the cells were resuspended. Cell lysis was done by sonication with a Bandelin MS 73 sonotrode on a 2070.2 pulse generator (Bandelin, Berlin, Germany). Sonication was performed twice for 5 min with 80% amplitude and pulse cycles with 2 s on, 2 s off, with a 5 min cooling phase between the sonication cycles.

Proteins with membrane anchors were solubilized after sonication by addition of 2 % (*v/v*) nonidet P-40 (Bachem Holding AG, Bubendorf, Switzerland) and rotation at 10 rpm in an overhead rotator for 1.5 to 2 h at room temperature.

After sonication (and solubilization) samples were centrifuged at 15,000× *g* for 45 min, 22 °C to pellet cell debris. The supernatant was used for IMAC. In case of eGFP-Vam3p’ the protein yield could be increased by performing a second extraction with N,N-dimethyldodecylamine-N-oxide (LDAO). Therefore, after decanting the supernatant and using it in IMAC, the pellet of cell debris was resuspended in the same volume of binding buffer with 3% (*v*/*v*) LDAO than had been decanted and rotated on an overhead rotator at 10 rpm and at room temperature overnight. The sample was then centrifuged again at 15,000× *g* as described above.

IMAC was done with an ÄKTA™ pure 25 system (Cytiva, Freiburg, Germany) using HisTrap crude FF colums (Cytiva, Freiburg, Germany). Columns were first equilibrated with IMAC binding buffer for 5 column volumes (CV) at 1 CV min^−1^, then applying the cleared cell lysate at 0.8 CV min^−1^. A washing step with IMAC binding buffer over 10 CV followed at a flow-rate of 1 CV min^−1^. When purifying membrane anchoring proteins, a second washing step over 10 CV was added, with a mixture of 87.5:12.5 IMAC binding buffer:IMAC elution buffer (20 mM sodium phosphate, 500 mM NaCl, 500 mM imidazole, pH 7.5). Protein was then eluted with IMAC elution buffer over 5 CV at a flow-rate of 0.7 CV min^−1^. For immobilization of membrane anchoring proteins buffer with high salt concentration was necessary. Therefore, no buffer exchange was performed after purification. Even without adding detergents to the IMAC elution buffer no aggregation of proteins with membrane anchors occurred over time periods of several weeks.

### 4.3. Polymersome Formation and Functionalization

Polymersomes were prepared in miniaturized stirred-tank reactors (bioREACTOR48, 2mag AG, Munich, Germany) as described by Poschenrieder et al. [21]. In brief, the unbaffled reactors were filled with 11.4 mL buffer with or without protein. An S-shaped stirrer was driven at 4,000 rpm. As soon as the stirrer had reached its speed, 0.6 mL of the 20% (*w*/*v*) PMOXA_15_-*b*-PDMS_68_-*b*-PMOXA_15_ block-copolymer (Polymer Source Inc., Dorval, Canada) solution in undenatured ethanol was injected with a syringe into the aqueous phase and stirred continuously. Progression of the polymersome formation process was checked in regular intervals by dynamic light scattering (DLS) measurements. When z-average and PDI were constant, usually after around 3 h, stirring was stopped. The temperature was controlled to 20 °C during the whole process.

Functionalization of polymersomes was either done during polymersome formation or by the immobilization of membrane anchoring proteins on the surface of preformed polymersomes. For the latter, polymersomes were mixed with protein and incubated overnight at room temperature. Non-functionalized protein was then separated by size-exclusion chromatography.

### 4.4. Dynamic Light Scattering (DLS)

For DLS measurements a ZetaSizer Nano S (Malvern Panalytical GmbH, Kassel, Germany) was used. Measurements were conducted as described previously [16]. Prior to the measurements, samples were diluted 1:10 with fresh buffer. The measurements were conducted at 25 °C.

### 4.5. Size-Exclusion Chromatography (SEC)

Size-exclusion chromatography (SEC) of polymersome samples was done with Sepharose 4B medium (Cytiva, Freiburg, Germany). Columns were packed to a height of 60 mm and had a bed volume of 12 mL. Tris buffer (20 mM tris-HCl, 100 mM NaCl, pH 8.0) was used as the mobile phase. SEC separation was done at room temperature (about 22 °C) and the mobile phase was allowed to flow through the column by gravity, resulting in a flow rate of around 11–12 cm/h.

### 4.6. Proteinase K Digest

Unspecific proteolytic digest of proteins on the outer surface of polymersomes was done by adding proteinase K (Carl Roth GmbH & Co. KG, Karlsruhe, Germany) to a concentration of 50 µg/mL and 5 mM CaCl_2_ to the polymersome dispersion. Samples were incubated at room temperature overnight.

### 4.7. Fluorescence and Light Extinction Measurements, Quantification of eGFP and Polymersome Concentrations

EGFP fluorescence and light extinction were measured in 96-well plates (black with clear bottom, Greiner Bio-One GmbH, Frickenhausen, Germany) in an Infinite M200 pro microplate reader (Tecan Group, Männedorf, Switzerland). Fluorescence of eGFP was measured at 485 nm excitation and 515 nm emission. Light extinction was measured in the absorption mode at 350 nm. Samples of functionalized polymersomes were measured after SEC. The concentrations of eGFP and eGFP-membrane anchor constructs were determined by comparison to individual fluorescence-standards. Due to the difference in eGFP fluorescence intensity between the individual proteins separate standard-curves with the respective protein were used. Separate standard-curves were also measured for samples without and with polymersomes. The protein concentrations derived from fluorescence intensity measurements and the polymersome concentrations resulting from light extinction measurements (350 nm) were taken to calculate the number of eGFP per polymersome.

### 4.8. Proteolysis with TEV Protease

TEV protease was added to the fusion proteins with TEV protease recognition sequences at a ratio of 1:20 TEV protease:protein based on the light absorption at 280 nm. Samples were incubated at the indicated temperature or room temperature (around 22 °C). For polymersome containing samples TEV protease was added in a 1:5 volumetric ratio (OD_280_ of the TEV protease solution was 1.5 at 10 mm path length), since polymersomes interfered with the light absorption measurement. Polymersomes were incubated with TEV overnight at room temperature and subsequent processed by SEC to separate polymersomes from proteolyzed protein.

### 4.9. Intein Splicing

To induce intein splicing, thiols were added to the purified protein at a concentration of 50 mM unless stated otherwise. Samples were incubated at room temperature (around 22 °C) or at the temperature specified.

## 5. Conclusions

In this study it was shown that hydrophobic membrane anchoring peptides can greatly enhance protein encapsulation in polymersomes. Fluorescence measurements revealed that the highest number of encapsulated proteins (eGFP) was achieved with the short and highly hydrophobic UBC6′ membrane anchor encapsulating up to 89.8 eGFP per polymersome. In comparison to statistical encapsulation of proteins, encapsulation was increased by 299.3 fold.

A comparison of four different membrane anchors—two with moderate and two with high hydrophobicity—showed that the more hydrophobic membrane anchors enabled a higher increase in protein encapsulation, but at the same time protein folding was affected. If only anchors are considered that have shown no negative effect on the folding of eGFP, 43.6 (eGFP-Cytb’5) and 39.4 (eGFP-L’) proteins were encapsulated per polymersome, which also represent strong improvements compared to statistical encapsulation. Next to the increase in encapsulation the choice of the appropriate membrane anchor should therefore also consider factors like protein expression titer and the effect of the anchor on protein folding.

Further, the targeted and specific release of immobilized proteins from the polymersome surface was investigated. Proteolysis with TEV protease and protein splicing of an introduced intein domain enabled the release of the immobilized eGFP domains from the vesicles. The direct comparison of both strategies showed that the protease digestion enabled a more complete removal of protein domains on the vesicle surface, while at the same time the encapsulation in the lumen was not impaired by the additional amino acids of the TEV cleavage site.

Currently, the production of functionalized polymersomes in a scalable process is an actively researched field. The availability of a method that increases protein encapsulation in a scalable polymersome manufacturing process is an important step towards the use of functionalized polymersomes in commercial products.

## Figures and Tables

**Table 1:** Dynamic light scattering measurements of polymersomes functionalized with membrane-anchoring eGFP. Errors represents the standard deviation in technical triplicates.

Sample	z-Average, nm	PDI, -	Protein Concentration, g/L	# eGFP per Polymersome on the Outer Surface
No protein	209.8 ± 1.7	0.224 ± 0.017	0.00	-
eGFP	217.2 ± 0.9	0.241 ± 0.004	0.50	-
eGFP-L’	246.7 ± 7.2	0.392 ± 0.018	0.49	158.4
eGFP-Cytb5′	251.2 ± 2.4	0.395 ± 0.049	0.50	114.3
eGFP-Vam3p’	237.2 ± 3.5	0.391 ± 0.016	0.50	44.9
eGFP-UBC6′	244.8 ± 2.8	0.375 ± 0.037	0.40	78.9

**Table 2:** Overview of the expression yield of the membrane-anchored eGFP variants per liter bacterial culture, their relative fluorescence compared to eGFP without membrane anchor, and their encapsulation efficiency at the loading optimum resulting in the highest number of encapsulated proteins. The suitability of the constructs is calculated as product (A × B × C). To get a dimensionless number, the expression yield was transformed into a weight/volume percentage concentration for this purpose.

Sample	A: Expression Yield, mg/L	B: Relative Fluorescence, %	C: EE%, %	Suitability (A × B × C) × 10^8^
eGFP-L’	5.50	103 ± 10 ^(^^a)^	5.87	33
eGFP-Cytb5′	3.10	103 ± 6 ^(^^a)^	6.00	19
eGFP-Vam3p’	3.85	27 ± 6 ^(^^a)^	25.68	27 ^(^^b)^
eGFP-UBC6′	0.96	7 ± 4	19.67	1.3 ^(^^b)^

^(a)^ Data from [22]; ^(b)^ Membrane anchors with significant effects on protein folding.

## Data Availability

The key data of the study are explicitly mentioned in the tables and in the text of the article. Additional data can be provided upon request.

## Supplementary Materials

The following are available online at https://www.mdpi.com/article/10.3390/ijms22137134/s1, Supplement S1—theoretical maximum of statistical encapsulation. Supplement S2—proteinase K digest of proteins immobilized on PMOXA-PDMS-PMOXA polymersomes. Supplement S3—protein encapsulation with hydrophobic membrane anchoring peptides. Supplement S4—diffusion of dithiothreitol (DTT) across the polymersome membrane. Supplement S5—effect of thiol concentration, temperature and the intein-preceding amino acid on intein splicing. Supplement S6—Oligonucleotides and sequences of synthetic genes.
