# Supplementary material for: Increased Protein Encapsulation in Polymersomes with Hydrophobic Membrane Anchoring Peptides in a Scalable Process
Source: Int J Mol Sci. 2021 Jul 1;22(13):7134. doi: 10.3390/ijms22137134 (PMC8268381; doi:10.3390/ijms22137134)
Supplement: Supplementary file 1 [file ijms-22-07134-s001.zip › ijms-1248355-supplementary.pdf]

Supplementary information to:

Increased protein encapsulation in polymersomes with hydrophobic membrane anchoring peptides in a scalable process

Michael Mertz [1] and Kathrin Castiglione [1,*]

Affiliation 1; Institute of Bioprocess Engineering, Friedrich-Alexander-Universität Erlangen-Nürnberg, Paul-Gordan-Straße 3, 91052 Erlangen, Germany
* Correspondence: kathrin.castiglione@fau.de

Supplement S1 – theoretical maximum of statistical encapsulation

Figure S1 gives an overview of the parameters important for the calculation of the number of eGFP molecules, which would cover the outer and the inner polymersome surfaces by a single protein layer. The available surface area was calculated from the surface of a sphere (A_P) according to equation (1), with r_P being the radius of the sphere / polymersome. The space required by each eGFP molecule is given by the area (A_{eGFP}) of a plane going through the center of the protein (equation (2)), resulting in the maximal occupied area for a globular protein. r_{eGFP} is the hydrodynamic radius of eGFP (2.3 nm [1]).

For the eGFP layer on the outer surface (outer protein layer, opl) the radius of an eGFP molecule (r_{eGFP}) is added to the radius of the polymersomes (r_P), giving the radius of the opl (r_{opl}, equation (3)). To calculate the molecules covering the inner surface, the radius of the inner protein layer (r_{ipl}) must be calculated. Therefore, the membrane thickness (d_{mem}) and r_{eGFP} were subtracted from r_P (equation (4)).

Given a tightest packing density of circles on a plain of 90.7 %, the number of eGFP molecules in single protein layers is 2252 for the outer surface and 1027 for the inner surface.

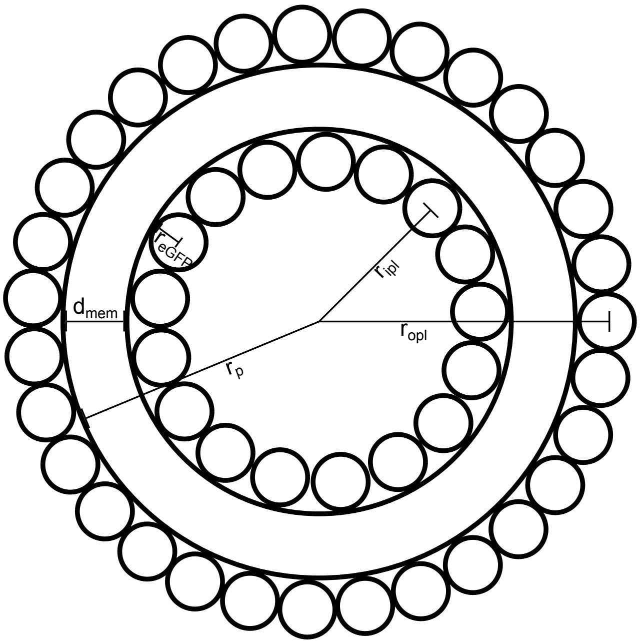

Figure S1. Schematic presentation of eGFP layers on the outer and inner surface of a polymersome. Small circles represent eGFP molecules, the large circles represent the outer- and inner border of the polymersomal membrane. The parameters important for the calculation of the number of eGFP molecules forming single protein layers on the outer- and inner surface are indicated. Explanation of the symbols is given in the text.

$$A_p = 4 * \pi * r_p^2, \quad (1)$$

$$A_{eGFP} = \pi * r_{eGFP}^2, \quad (2)$$

$$r_{opl} = r_p + r_{eGFP}, \quad (3)$$

$$r_{ipl} = r_p - (d_{mem} + r_{eGFP}), \quad (4)$$

Radius eGFP: r_{eGFP} = 2.3 nm [1]

Diameter membrane: d_{mem} = 14 nm [2]; the thickness of the PMOXA-PDMS-PMOXA polymer membrane (d_{mem}) had been determined by electron microscopy.

Diameter polymersome: d_P = 110 nm [3]; the average diameter of the PMOXA-PDMS-PMOXA polymersomes had been determined by dynamic light scattering measurements of unfunctionalized polymersomes. To compensate for the higher intensity of the scattered light from larger particles the number based average diameter (n-average) had been used.

Supplement S2 – proteinase K digest of proteins immobilized on PMOXA-PDMS-PMOXA polymersomes

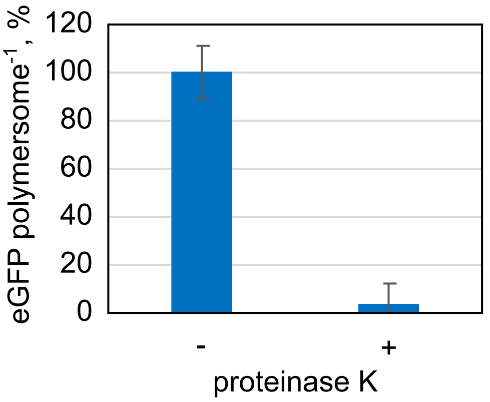

Figure S2. Quantification of the removal of immobilized eGFP from the polymersome surface. eGFP-Cytb5′ were immobilized on the outside of polymersomes and incubated with proteinase K (50 µg mL^{-1}) overnight at room temperature. The standard deviation in technical triplicates is indicated by the error bars.

Supplement S3 – protein encapsulation with hydrophobic membrane anchoring peptides

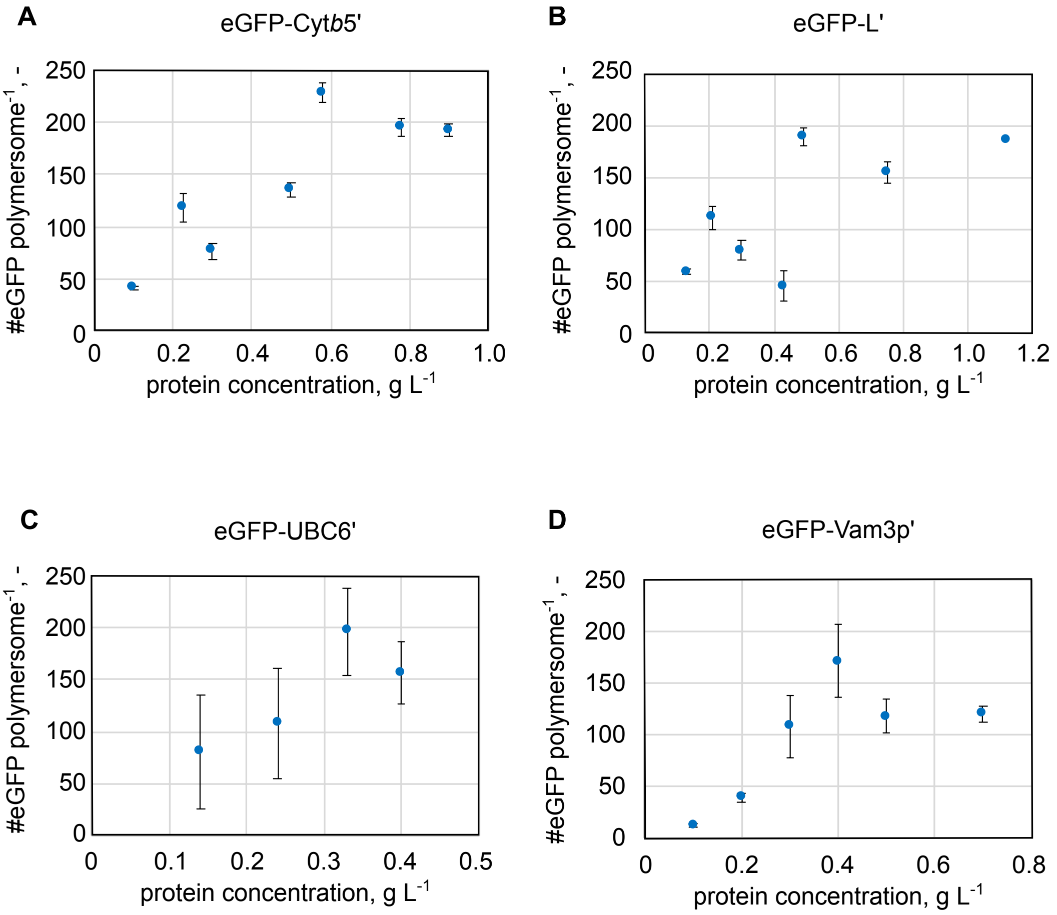

Figure S3. Functionalization of polymersomes with fusion proteins of enhanced green fluorescent protein (eGFP) with membrane anchors from cytochrome b_5 (Cytb5′), the lysis protein L (L′), the ubiquitin conjugating enzyme 6 (UBC6′) and the syntaxin Vam3p (Vam3p′). Polymersomes were prepared in presence of varying protein concentrations. After formation, polymersomes were separated from unencapsulated protein by SEC. Concentrations of eGFP and polymersomes contained in the samples were determined from the intensity of eGFP fluorescence and optical density at 350 nm resulting from

light scattering by the polymersomes. From the comparison to fluorescence intensity (individual for each protein) and light-scattering intensity standards the number of encapsulated eGFP molecules per polymersome was calculated. Error bars indicate the standard deviation in technical triplicates.

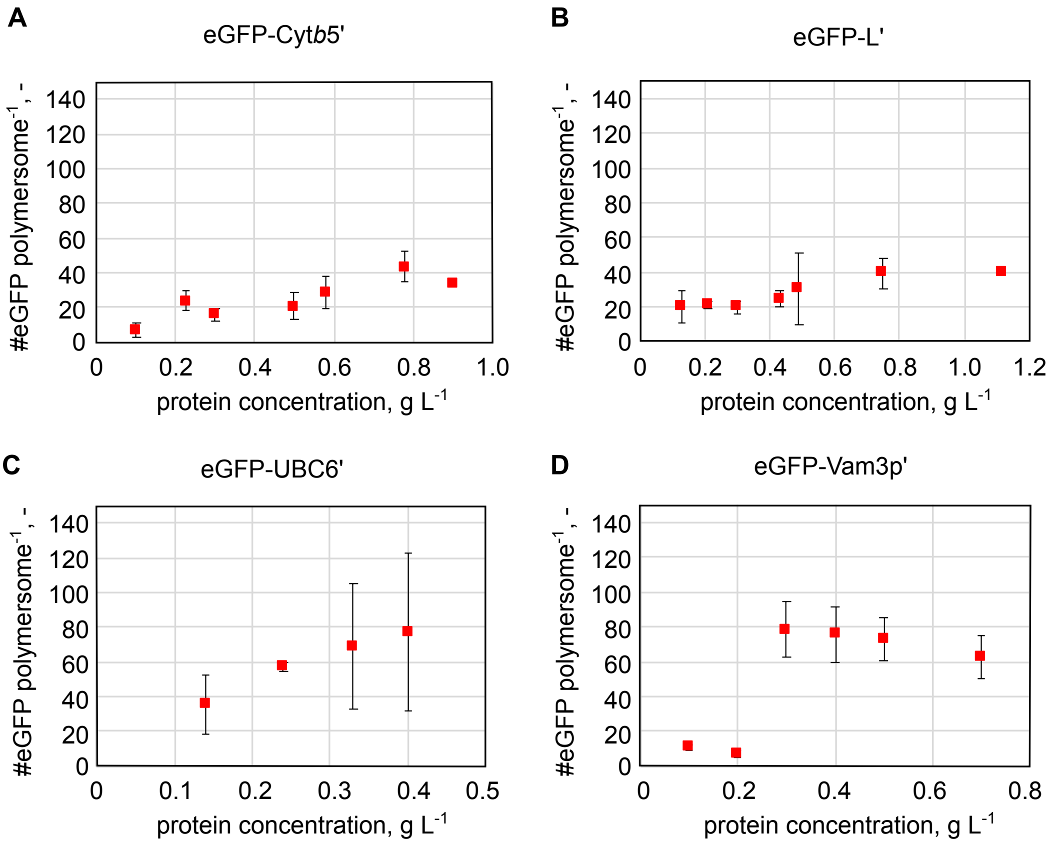

Figure S4. Number of encapsulated eGFP molecules per polymersome after formation in presence of varying protein concentrations. Samples are corresponding to Figure S3. Samples of polymersomes as prepared for the analysis shown in figure S3 were then treated with proteinase K, followed by SEC. Then final measurement of the eGFP fluorescence intensity and optical density at 350 nm was again used to derive the number of proteins per polymersome. The standard deviation of technical triplicates is indicated by the error bars.

Supplement S4 – diffusion of dithiothreitol (DTT) across the polymersome membrane

DNTB assay

For the quantification of thiols the DNTB (5,5′-dithio-bis-(2-nitrobenzoic acid)) assay was used [4]. For this purpose, 140 µL of samples containing polymersomes encapsulating thiols or TECEP (tris(2-carboxyethyl)phosphine) as a control were placed into a clear 96 well plate (Brand GmbH & Co. KG, Wertheim, Germany), then adding 140 µL of the DNTB reagent solution (4 mg mL^{-1} DNTB in 100 mM sodium phosphate, 1 mM EDTA, pH 8.0) and immediately starting the absorption measurement at 412 nm over 30 min in a Tecan Infinite® M200 pro microplate reader (Tecan group, Männedorf, Switzerland). Substances were quantified via previously recorded standards

To probe diffusion of thiols across the membrane of PMOXA$_{15}$-PDMS$_{68}$-PMOXA$_{15}$ polymersomes, different experimental designs are possible. For slowly diffusing substances, a so-called Influx Assay has been established and used to determine permeability coefficients of aminosugars, caffeine, pyruvate and nucleotides on these polymersomes [5]. The assay is based on the formation of empty polymersomes, which are then incubated in solutions of the investigated substance for a set time. After the incubation-time polymersomes are separated from the

diffusing substance by size exclusion chromatography (SEC). After lysis of the polymersomes the amount of the investigated substance, which diffused during the time of incubation is quantified.

While this works well for substances diffusing slowly, in relation to the time needed for SEC (roughly 5 to 7 minutes for the elution of polymersomes), for fast diffusing substances other experimental designs should be evaluated. A schematic representation is given in Figure S5 A, where the chemical used for the quantification assay is encapsulated. The non-encapsulated molecules are removed by SEC, then adding thiols over a range of concentrations and directly measuring the diffusion of thiols into the polymersomes. This has the prerequisite, however, that the quantification reagent (DNTB, also known as Ellman's reagent) cannot diffuse over the polymersome membrane. Previous studies showed on the one hand that the lipid membrane of liposomes acted as a diffusion barrier for DNTB [6]. On the other hand significant transfer of DNTB over the polymer membrane of poly(ethylene oxide)-poly(butylene oxide) polymersomes had been observed [7]. Therefore, a preliminary test was conducted, which suggested that DNTB did diffuse over the membrane of PMOXA-PDMS-PMOXA polymersomes (data not shown). Therefore, the second experimental setup (Figure S5 B) was taken into account. It was based on the encapsulation of the thiol, subsequent removal of non-encapsulated thiol by SEC and the quantification of remaining thiol via DNTB. Since the thiol in question, dithiothreitol (DTT), was expected to diffuse fast, this experiment can show diffusion of the thiol over the polymer membrane during the (short) time needed for SEC (5 to 7 minutes) by reduction of remaining thiol. However, no quantitative results can be obtained.

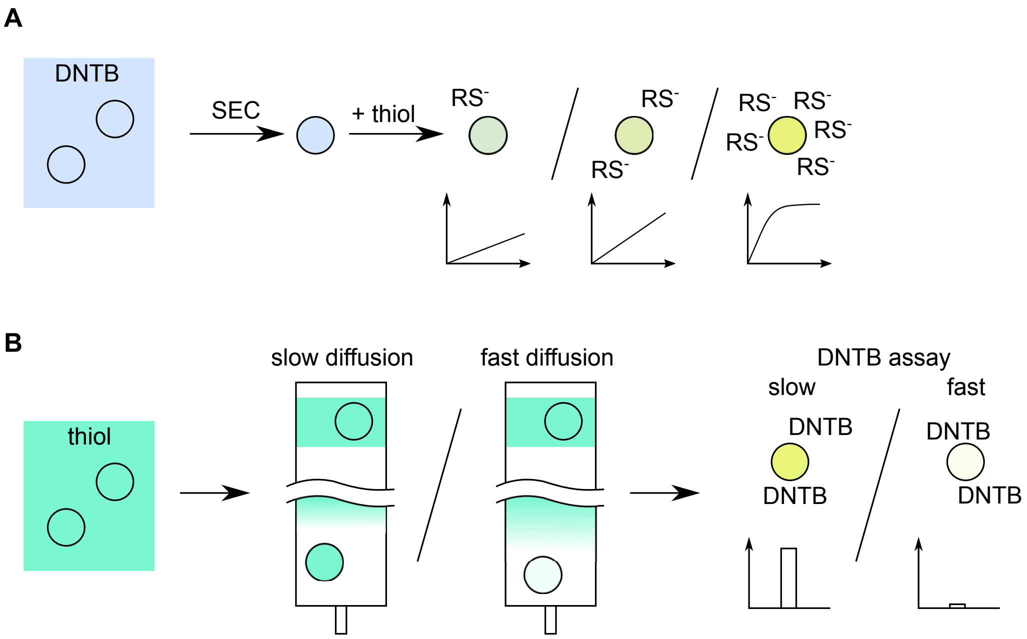

Figure S5. Schematic representation of experimental setups used to probe thiol diffusion across the polymer membrane of polymersomes. A: Encapsulation of the reagent used for quantification (DNTB) to measure diffusion of thiols into polymersomes directly. B: encapsulation of thiols, followed by separation of non-encapsulated thiols by size-exclusion chromatography (SEC), starting the diffusion phase and quantification of remaining thiol after SEC.

The assay described above and in Figure S5 B was done with DTT, cysteine for comparison and TECEP (tris(2-carboxyethyl)phosphine) as a control, since another study showed lipid membranes to present a high diffusion barrier for TECEP [6]. After SEC, polymersomes were measured in the DNTB assay, which showed almost no remaining DTT (2.8 %), 52.4 % of cysteine and 88.1 % of TECEP (Figure S6). This indicated, that DTT diffused rather rapidly, in seconds or minutes over the PMOXA-PDMS-PMOXA polymer membrane, followed by the slower diffusion of cysteine and TECEP. These results are in agreement with the previous studies showing diffusion of DTT over cellular membranes [8], slow to no diffusion of TECEP over lipid

membranes [6] and slower diffusion of charged molecules over the polymer membrane of PMOXA-PDMS-PMOXA polymersomes [5].

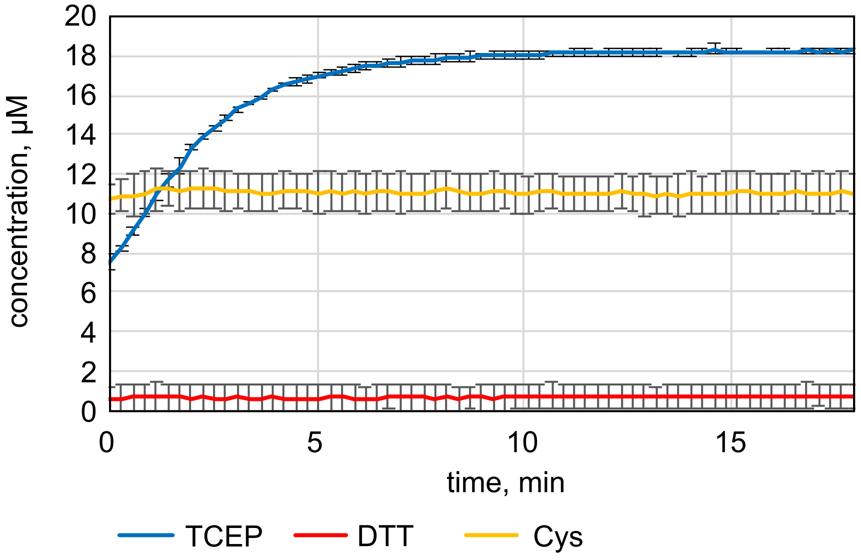

Figure S6. Quantification of the remaining thiols in polymersomes by the DNTB assay of polymersomes after encapsulation of DTT, cysteine and TECEP and removal of the non-encapsulated thiols by SEC. Error bars indicate the standard deviation in technical triplicates.

Supplement S5 – effect of thiol concentration, temperature and the intein-preceding amino acid on intein splicing

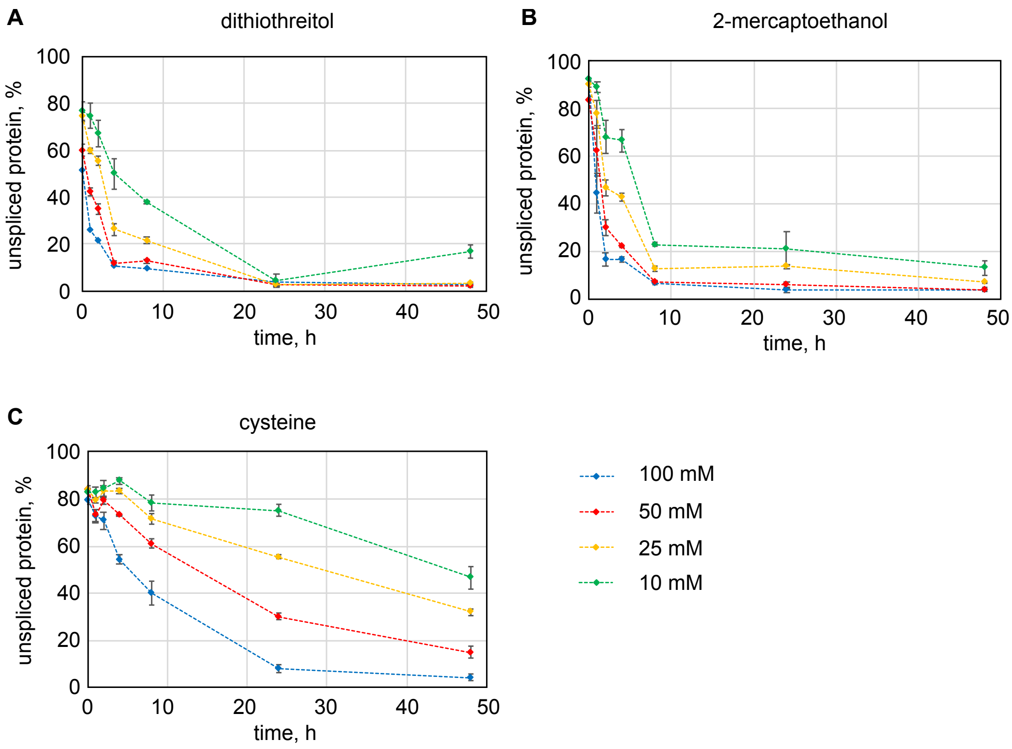

Figure S7. Splicing of the fusion protein eGFP-Int-Cytb5′ with dithiothreitol (A), 2-mercaptoethanol (B) and cysteine (C) at thiol concentrations between 10 and 100 mM. The error bars show the standard deviation in technical triplicates.

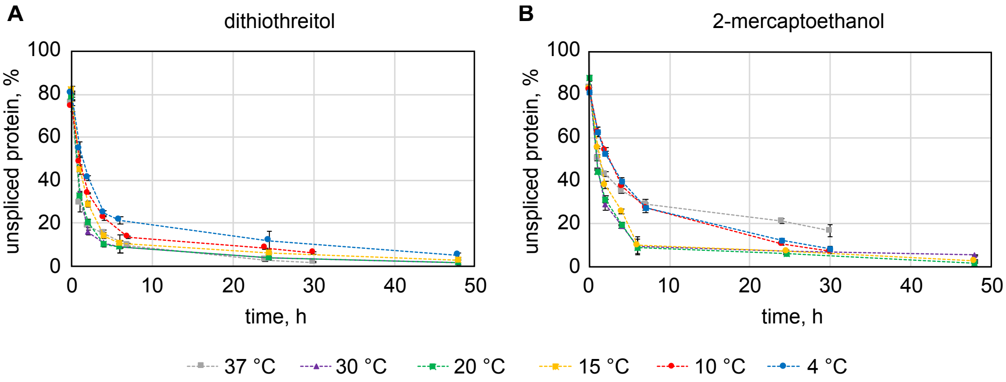

Figure S8. Progression of intein splicing of eGFP-Int-Cytb5′ with 50 mM DTT (A) or 2-mercaptoethanol (B) at temperatures between 4 and 37 °C. Standard deviation in technical triplicates is indicated by the error bars.

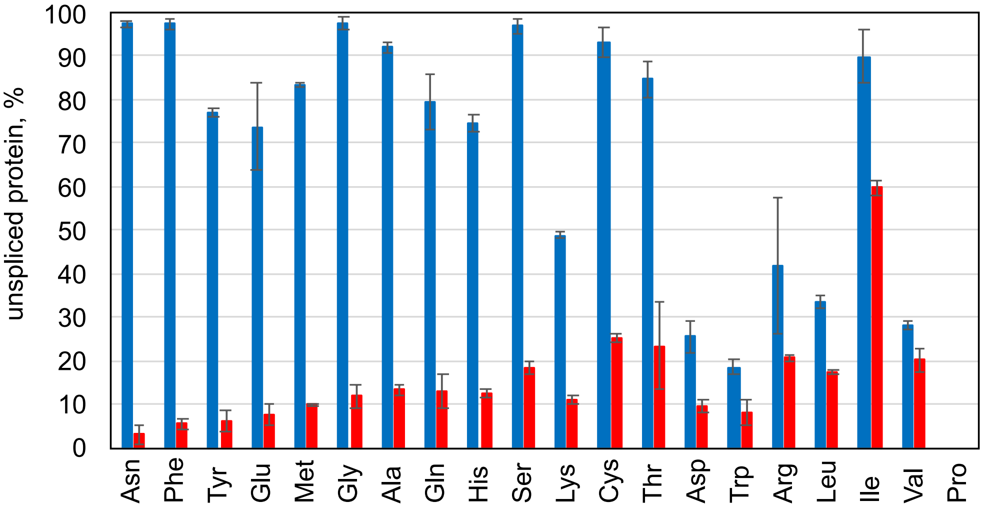

Figure S9. Influence of the amino acid preceding the intein domain (-1 position) on thiol induced intein splicing. After protein purification the eGFP-Int(-1X)-pA-Cytb5′ was incubated at room temperature overnight with (red bar) or without (blue bar) 50 mM DTT. The distribution between spliced and unspliced protein as analyzed by SDS-PAGE. The height of the bars represents the amount of unspliced protein found after overnight incubation. The deviation found in three samples handled in parallel is represented by the error bars.

Supplement S6 – Oligonucleotides and sequences of synthetic genes

Table S1. Oligonucleotides used for cloning of genetic constructs used in this study.

oligonucleotide name	Sequence, 5′ to 3′
eGFP NdeI F	GATACACATATGGTGAGCAAGGGCGAGGAG
GSGG2 EcoRI R	TGATGAATTCACCGGAACCACCGCCAGAGCC ACCGCCGGATCCCTT GTACAGCTCGTCCATGCC
AOC GS1 F	GATCCGGCAGCTCTTCCTCGGAAAACCTGTA TTTT CAGAGTGGTTCGTCCTCTAGTGC
AOC GS1 R	GGCCGCACTAGAGGACGAACCACTCTGAAA ATACAGGTTTT CCGAGGAAGAGCTGCCG
AOC EA1 F	GATCCGAGGCTGCAGCCAAGGAGAACCTGTA TTTT CAGAGCGAAGCTGCGGCCAAAGC
AOC EA1 R	GGCCGCTTTGGCCGCAGCTTCGCTCTGAAAA TACAGGTTCTCCTTGGCTGCAGCCTCG
EA2 TEV BamHI F	GCAACATCCTGGGGC
EA2 TEV NotI R	TCAATGCGGCCGCTTTCGCTGCCGCTTCTTTG
EA3 TEV BamHI F	TCATAGGATCCGAGGCCGC
EA3 TEV NotI R	CCTTTGCGCGACGTGCGG
GS2 TEV BamHI F	TAAGATGGATCCGGCTC
GS2 TEV NotI R	TCAATGCGGCCGCGCTGCTGCTACTCCCAC
GS3 TEV BamHI F	GGCCACCTCTTGGGGATCC
GS3 TEV NotI R	AAGTAGCGGCCGCGCTGC
Int Y-1X F	GTACAAGGGATCCNNNTGCATCACAGGGGA TG
Int Y-1X R	GGATCCCTTGTACAGCTCGTC

Table S2. Sequences of synthetic DNA used in the genetic constructs produced in this study.

Gene / sequence name	Sequence
Mycobacterium xenopi gyrase A intein	GGATCCTATTGCATCACAGGGGATGCACTCGTTGCGTT A CCCGAAGGCGAAAGTGTGCGTATTGCCGATATCGTCC CTGGAGCTCGTCCGAACAGCGACAACGCCATTGACCT GAAAGTGCTTGATCGTCATGGCAATCCGGTACTTGCG GATCGCTTGTTTCACTCGGGTGAACATCCGGTCTACA CGGTACGCACAGTGGAAGGACTGCGCGTTACGGGCA CTGCCAATCATCCGCTCTTATGCCTGGTCGATGTGGC GGGTGTTCCGACCCTGTTGTGGAAGCTGATCGACGAG ATCAAACCGGGCGATTATGCGGTGATTCAGCGTTCTG CGTTTAGCGTGGATTGTGCGGGCTTTGCACGTGGCAA ACCGGAGTTTGCACCTACCACCTATACGGTCGGTGTT CCCGGTCTGGTGCGCTTTCTGGAAGCTCACCATCGCG ATCCAGACGCACAAGCCATTGCGGACGAACTGACCGA TGGTCGGTTCTACTACGCGAAAGTTGCCTCAGTCACC GATGCAGGGGTTCAGCCAGTGTATTCGCTGCGCGTA GACACTGCGGATCACGCCTTCATTACCAACGGCTT CGTGTCCCATGCTGCGGCCGCGGCTGCGGCAGCTGCAG CT GCGGAATTC

GSSSS2-TEV-GSSSS2 and EAAAK2-TEV-EAAAK2	TAAGATGGAT CCGGCTCGTC AAGCTCAGGC AGCTCTTCCTC GGAAAACCTGT ATTTTCAGAGT GGTTCGTCCTC TAGTGGGAGTA GCAGCAGCGAA TTCGCAACATC CTGGGGCGGAT CCGAAGCCGCA GCGAAAGAGGC TGCAGCCAAGG AGAACCTGTAT TTTCAGAGCGA AGCTGCGGCCA AAGAAGCGGCA GCGAAAGAATT CAGTACTA
GSSSS3-TEV-GSSSS3 and EAAAK3-TEV-EAAAK3	TAAGATGGAT CCGGCTCGTC AAGCTCAGGCA GCTCTTCCTCG GAAAACCTGTA TTTTCAGAGTG GTTCGTCCTCT AGTGGGAGTAG CAGCAGCGAAT TCGCAACATCC TGGGGCGGATC CGAAGCCGCAG CGAAAGAGGCT GCAGCCAAGGA GAACCTGTATT TTCAGAGCGAA GCTGCGGCCAA AGAAGCGGCAG CGAAAGAATTC AGTACTA

References

1. Hink, M.A.; Griep, R.A.; Borst, J.W.; Van Hoek, A.; Eppink, M.H.; Schots, A.; Visser, A.J. Structural dynamics of green fluorescent protein alone and fused with a single chain Fv protein. *J. Biol. Chem.* **2000**, *275*, 17556-17560.
2. Poschenrieder, S.T.; Wagner, S.G.; Castiglione, K. Efficient production of uniform nanometer‐sized polymer vesicles in stirred‐tank reactors. *J. Appl. Polym. Sci.* **2016**, *133*.
3. Klermund, L.; Poschenrieder, S.T.; Castiglione, K. Biocatalysis in polymersomes: improving multienzyme cascades with incompatible reaction steps by compartmentalization. *ACS Catalysis* **2017**, *7*, 3900-3904.
4. Ellman, G.L. A colorimetric method for determining low concentrations of mercaptans. *Arch. Biochem. Biophys.* **1958**, *74*, 443-450.
5. Poschenrieder, S.T.; Klermund, L.; Langer, B.; Castiglione, K. Determination of permeability coefficients of polymersomal membranes for hydrophilic molecules. *Langmuir* **2017**, *33*, 6011-6020.
6. Cline, D.J.; Redding, S.E.; Brohawn, S.G.; Psathas, J.N.; Schneider, J.P.; Thorpe, C. New water-soluble phosphines as reductants of peptide and protein disulfide bonds: reactivity and membrane permeability. *Biochemistry* **2004**, *43*, 15195-15203.
7. Battaglia, G.; Ryan, A.J.; Tomas, S. Polymeric vesicle permeability: a facile chemical assay. *Langmuir* **2006**, *22*, 4910-4913.
8. Le Gall, S.; Neuhof, A.; Rapoport, T. The endoplasmic reticulum membrane is permeable to small molecules. *Molecular biology of the cell* **2004**, *15*, 447-455.
